# Targeted DNA Nanomachine Enables Specific miRNA‐Responsive Singlet Oxygen Amplification for Precise Cutaneous Squamous Cancer Therapy

**DOI:** 10.1002/advs.202415296

**Published:** 2025-03-21

**Authors:** Hanane Aliouat, Detian Zhang, Lanyuan Peng, Jiaxin Huang, Hongshi Cheng, Jiaojiao Zhu, Xiang Chen, Nuli Xie, Wenhu Zhou, Shuang Zhao

**Affiliations:** ^1^ Xiangya School of Pharmaceutical Sciences Central South University Changsha Hunan 410013 China; ^2^ Department of Dermatology Hunan Engineering Research Center of Skin Health and Disease Hunan Key Laboratory of Skin Cancer and Psoriasis Xiangya Hospital Central South University Changsha Hunan 410008 China; ^3^ Hangzhou Innovation Institute Beihang University Hangzhou Zhejiang 310053 China

**Keywords:** DNA nanotechnology, DNA tetrahedra, microRNA, photodynamic therapy, skin tumors

## Abstract

Photodynamic therapy (PDT) is a promising strategy for the treatment of skin‐related tumors including cutaneous squamous cells carcinoma (cSCC). However, it is hard to balance the dosage off‐target phototoxicity while maintaining satisfactory therapeutic effect. In addition, oxygen‐dependent photosensitizers (PSs) are affected by tumor hypoxic environment, which further causes inefficient photocatalysis and reduces therapeutic effect. Herein, an intelligent DNA nanomachine based on tetrahedral DNA framework is proposed, incorporated with tumor‐targeted aptamer and specific miRNA‐responsive hairpin DNA catalytic assembly (HCA), for precise and high‐efficient therapy of cSCC. After aptamer‐mediated targeted delivery, a cSCC‐specific miRNA selected by tissue sequencing analysis is used to activateHCA, for amplifying PSs and controllably releasing chemotherapeutic drugs. Sequential recognition can greatly improve tumor‐specific accumulation and high‐dose activation. Moreover, hemin is incorporated into DNA to catalytically produce oxygen. In vitro and in vivo experiments demonstrated that this DNA nanomachine greatly improved anti‐tumor effect and realized effective ablation of cSCC in mice, with barely systemic toxicity and inflammation. It is anticipated that this strategy will promote biomedical applications of tumor‐specific miRNA and provide a promising option for the non‐invasive treatment of skin‐associated tumors.

## Graphical abstract

An intelligent DNA nanomachine based on DNA tetrahedron is proposed, incorporated with aptamer and specific miRNA‐responsive hairpin catalytic assembly (HCA), for precise therapy of cutaneous squamous cell carcinoma (cSCC) in mice, with barely systemic toxicity and inflammation. A cSCC‐specific miRNA is selected by RNA sequencing and bioinformatics analysis. Sequential recognition greatly improve tumor‐specific accumulation and HCA‐based photosensitizer amplification allows high‐efficient killing.

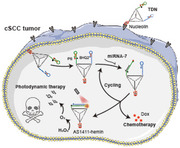

## 1 Introduction

Cutaneous squamous cell carcinoma (cSCC) is the second most common skin malignancy after basal cell carcinoma.^[^ ^1^, ^2^, ^3^ ^]^ Although surgical therapy is still the main therapeutic strategy, the excision of sensitive areas such as fingers, cheeks, lips and eyelids is usually hindered by loss of function and the unaesthetic outcome. In addition, the recurrence and aggressive features require frequent surgeries, holding huge barriers toward clinical management.^[^ ^4^ ^]^ Therefore, there is an urgent need to develop non‐invasive treatment modalities for cSCC. Photodynamic therapy (PDT) is an emerging strategy for non‐invasive tumor therapy, especially suitable to skin‐related tumors. It relies on photosensitizers (PSs) able to produce cytotoxic reactive oxygen species (ROS) upon light irradiation, leading to the disruption of cellular structure and apoptosis.^[^ ^5^, ^6^, ^7^ ^]^ However, a low dose is difficult to guarantee effective treatment. In addition, low intracellular delivery efficiency of PSs and tumor hypoxic microenvironment further hinder PDT effect. Due to the lack of targetability of the PS itself, a high dose can easily result in systemic distribution, ultimately leading to photophobia and uncontrollable phototoxicity.^[^ ^8^, ^9^, ^10^ ^]^ Thus, the strategies of tumor‐targeting and PS controlled activation have become a wise choice for PDT applications.

In recent years, benefiting from predictable Watson–Crick base pairing rule and excellent structural programmability, framework nucleic acids (FNA) have become an unparalleled platform for the construction of artificial nanodevices and nanomachines, showing great applications in biosensing,^[^ ^11^, ^12^ ^]^ cell biology,^[^ ^13^, ^14^ ^]^ and diseases therapeutics.^[^ ^15^, ^16^, ^17^, ^18^ ^]^ Among them, DNA tetrahedra are widely used in drug delivery and cancer therapy, thanks to its good loading capacity and structural rigidity.^[^ ^19^, ^20^ ^]^ Moreover, caveolin‐mediated transmembrane capacity allows effective intracellular delivery of the payloads, which are carried by covalent or non‐covalent modification.^[^ ^21^, ^22^, ^23^ ^]^ To achieve stimulus response such as acidity, RNA, and enzymes, FNAs are designed to incorporate functional molecules including aptamers and DNAzymes to construct responsive DNA nanomachines with single or multiple functions.^[^ ^24^, ^25^, ^26^ ^]^ For example, Lin's group^[^ ^27^ ^]^ designed HER2‐targeted DNA‐aptamer‐modified DNA tetrahedron as a drug delivery system for targeted delivering maytansine to HER2‐positive cancer.

MicroRNAs (miRNAs) are a class of short, non‐coding oligonucleotides that are widely involved in post‐transcriptional regulation of gene expression in cells.^[^ ^28^, ^29^ ^]^ The abnormal expression levels of miRNAs are closely implicated in cellular disfunctions, as they are related to protein synthesis, cell proliferation, apoptosis and other life activities. Nowadays, miRNAs have been recognized as important biomarkers for many diseases and tumors. A number of DNA‐based drug delivery systems have been developed to achieve controlled drug activation and release in response to tumor‐associated miRNA molecules.^[^ ^30^, ^31^ ^]^ Taking PSs as an example, the inactivation of PSs could be achieved through adjacent quenching molecules, and the phototoxicity in non‐target sites can be solved through the controllable activation.^[^ ^32^ ^]^ Nevertheless, for the case of miRNAs responsive systems, the amount of miRNA inside tumor cells remains very low even at high expression (equal to or below the pm level), which can lead to inadequate activation and insufficient drug releasing.

**Scheme 1:**
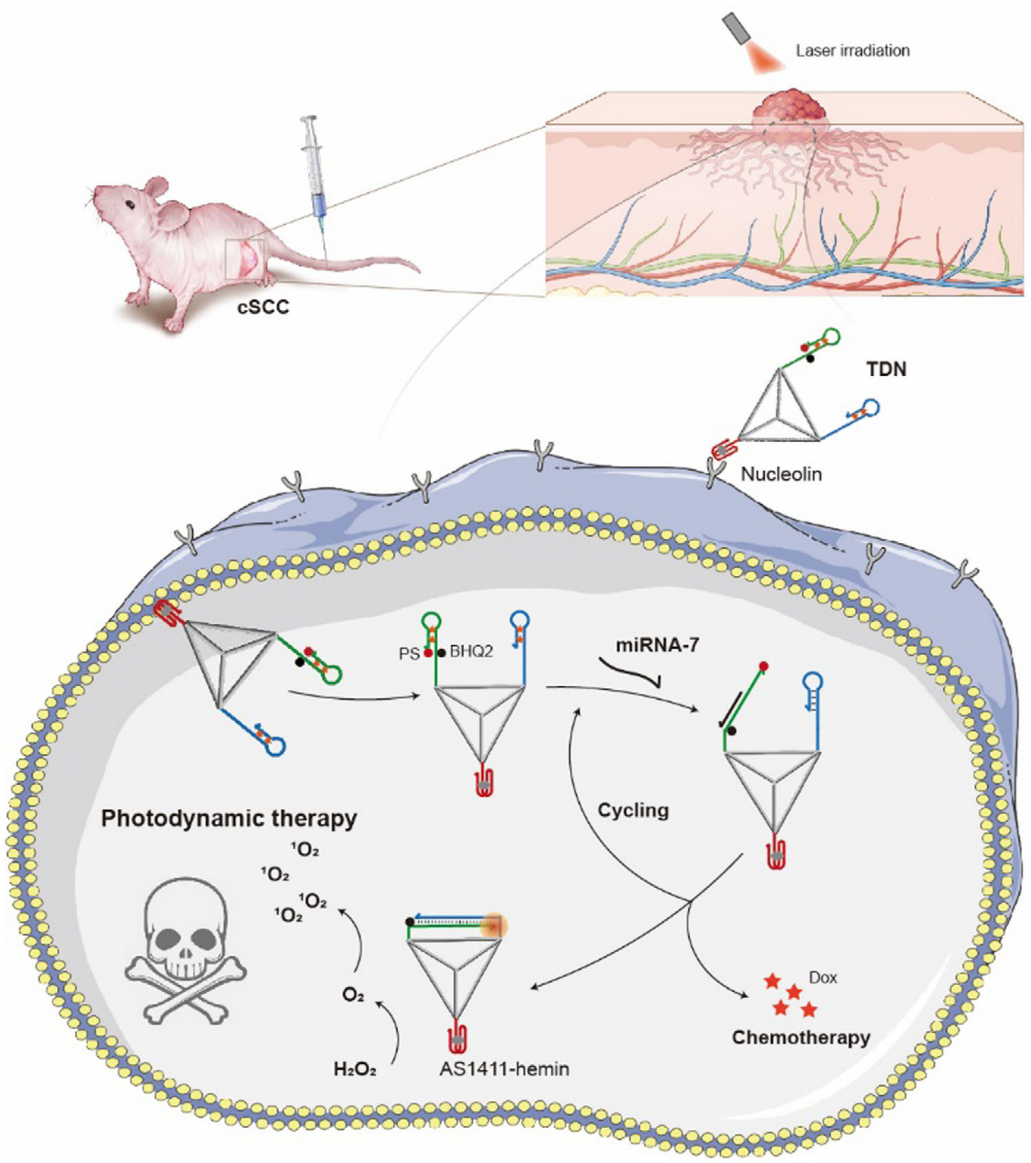
Schematic diagram of the working principle of TDN precisely targeting nucleolin protein overexpressed on the surface of tumor cell membrane and specifically responding to miRNA to achieve combined photodynamic and chemotherapy in cSCC tumor.

**Figure 1:**
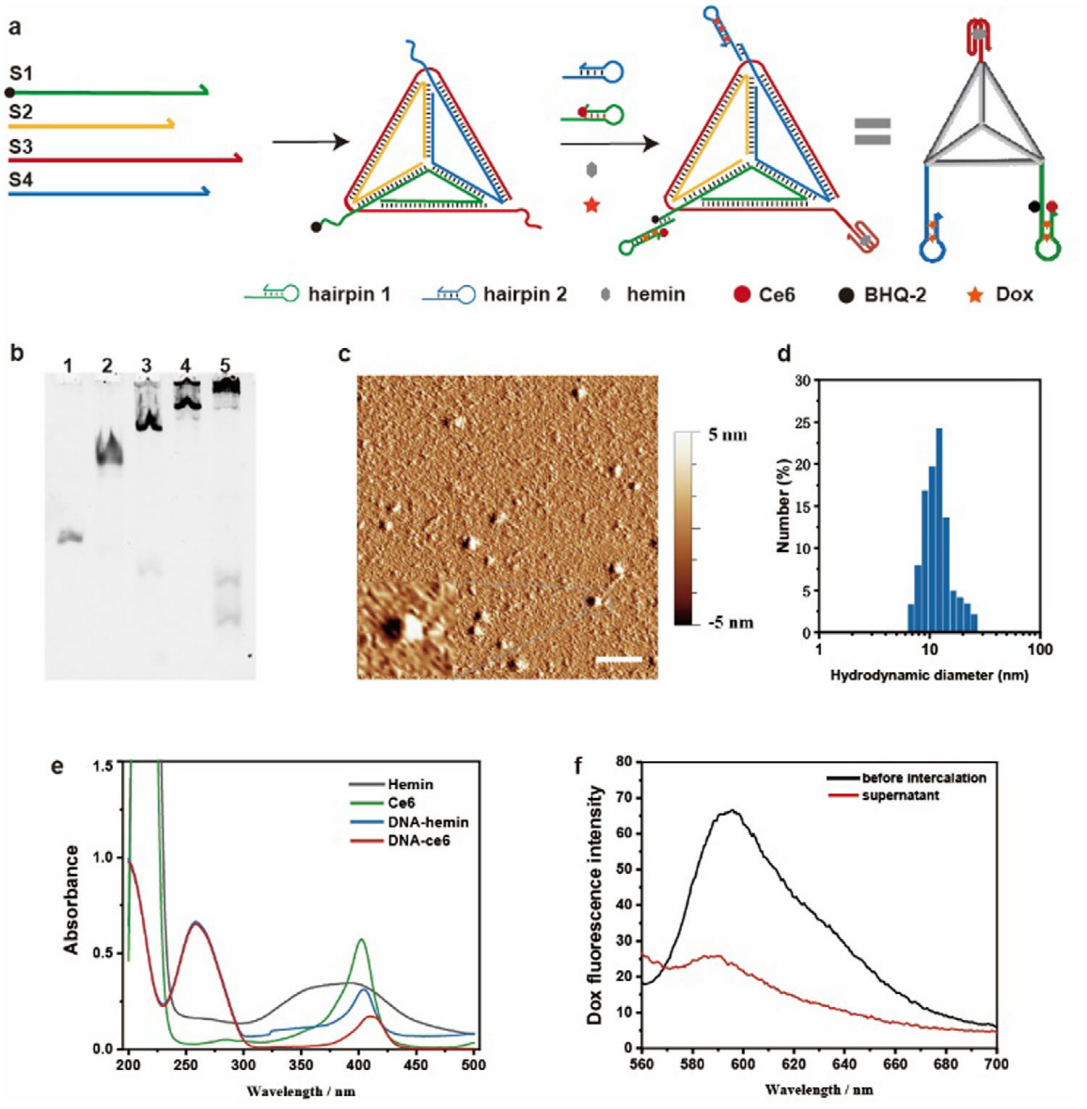
Preparation and characterization of TDN. a) Illustration scheme of the self‐assembly process of TDN. DNA tetrahedral framework was first assembled by four long strands. The hairpin and other drug molecules were then sequentially loaded onto the structure to form the final TDN. b) PAGE characterization of the TDN. Lane 1: S1, lane 2: S1 + S2, lane 3: S1 + S2 + S3, lane 4: S1 + S2 + S3 + S4, lane 5: S1 + S2 + S3 + S4 + H1 + H2. c) AFM characterization of the TDN. The scale bar is 50 nm. d) DLS characterization of the TDN. e) UV–vis spectroscopy results of the hemin and ce6 before and after loading into the TDN. f) Fluorescence spectroscopy of the Dox molecules in the supernatant after ultrafiltration, compared to that in solution.

To solve this problem, we developed an intelligent tetrahedra DNA nanomachine (TDN) targeting cSCC tumor and specifically responding to miRNA to achieve activation and amplification of PSs and sustained output of ROS under light irradiation (**Scheme** **1**). TDN was based on a tetrahedral DNA framework formed by the self‐assembly of four long oligonucleotides (**Figure** **1**a). AS1411 aptamer sequence was embedded at the end of strand S3, and formed a ligand structure at the vertex of DNA tetrahedra, enabling it to actively target tumors. Through hybridization, two hairpin DNA molecules were attached to two other vertices. Hairpin H1 activated HCA reaction in response to tumor‐specific miRNAs, then triggered the separation of PS from the adjacent quencher, resulting in its activation. Subsequently, due to the competitive strand displacement reaction of hairpin H2, the miRNA molecule was re‐released to open more hairpin molecules, thereby amplifying the activated dose of PSs. Considering the hypoxic microenvironment at the tumor site, the hemin molecule was inserted in the guanine‐rich aptamer, and the complex manifested catalase activity, able to catalyze hydrogen peroxide to oxygen. Furthermore, to enhance the killing effect on tumor tissues, doxorubicin molecules (Dox) were incorporated into the double‐stranded stem region of Hairpin H1 to achieve miRNA responsive release and synergistic therapy. Based on this strategy, DNA nanomachines carrying PSs and Dox can accumulate at the tumor site, realize miRNA‐responsive singlet oxygen amplification and controlled release of chemotherapy drugs, achieving the precise and high‐efficient treatment of cSCC.

## 2 Results and Discussion

### 2.1 Construction and Characterization of TDN

DNA tetrahedral nanostructures were constructed according to the previous annealing protocol reported in the literature.^[^ ^33^, ^34^ ^]^ After the DNA tetrahedron was assembled, two pre‐annealed hairpins H1 and H2 were mixed with the structures and reacted overnight, to form a stable DNA nanomachines by hybridizing to the extended sequence of tetrahedral vertices. The successful assembly of DNA nanostructure was verified by polyacrylamide gel electrophoresis (PAGE) (Figure 1b). With the increase of DNA strands, the band mobility of the corresponding lanes slowed down. Subsequently, we used atomic force microscopy (AFM) to characterize the morphology of TDN (Figure 1c). The results showed that the DNA nanomachines were tetrahedral structures with a relatively uniform size, and their height under AFM was ≈5 nm (Figure S1, Supporting Information), which was basically consistent with the theoretical outcome. The TDN was also analyzed by dynamic light scattering (DLS) (Figure 1d). Its hydrodynamic size distribution in buffer was ≈10 nm. The overall result demonstrated that DNA nanomachines were successfully assembled.

The PS carried by TDN is Chlorin e6 (Ce6), an organoporphyrin small molecule able to absorb light energy at a specific wavelength and produce reactive oxygen species and oxidative stress. Ce6 was modified onto the hairpin H1 by conjugation reaction, which effectively improved its water solubility. The UV–vis spectroscopy of the DNA‐ce6 complex showed a characteristic peak of ce6 at 410 nm, as well as a peak at 260 nm (Figure 1e). This indicated that ce6 was successfully modified onto the DNA strand. The Hemin molecules were embedded into the AS1411 aptamer that forms the G quadruplex structure. Similarly to DNA‐ce6, we verified the formation of the composite by UV–vis spectroscopy (Figure 1e). To enhance the therapeutic effect on skin tumors, DNA nanomachines can also carry Dox to implement chemotherapy. Specifically, Dox was embedded into the double‐stranded stem region of hairpins before constructing TDN. As expected, the fluorescence intensity in the supernatant after ultrafiltration decreased significantly, indicating that doxorubicin could be successfully loaded into DNA haripins (Figure 1f).

Notably, the key elements of HCA reaction are two hairpin molecules, H1 and H2. To ensure that the reaction can occur in a controlled manner, we used the NUPACK tool to screen and validate nucleic acid sequences (Figure S2, Supporting Information). The electrophoresis results showed that the two hairpins remained stable in the absence of a target, and the HCA reaction occurred only in the presence of the target (Figure S3, Supporting Information). In addition, since the PS is modified on the hairpin H1, the quencher is modified on the S1 strand hybridized to H1. In order to adequately inhibit the phototoxicity of PS when it is not activated, the molar ratio of the hairpin molecules and DNA tetrahedron nanostructures was optimized (Figure S4, Supporting Information). When the molar ratio is 1.5, a good balance between the activation and quenching states of the PS was achieved, ensuring that it would not be toxic to the body when it is not activated.

### 2.2 Selection of cSCC‐Specific miRNA and miRNA‐Responsive PDT Amplification

**Figure 2:**
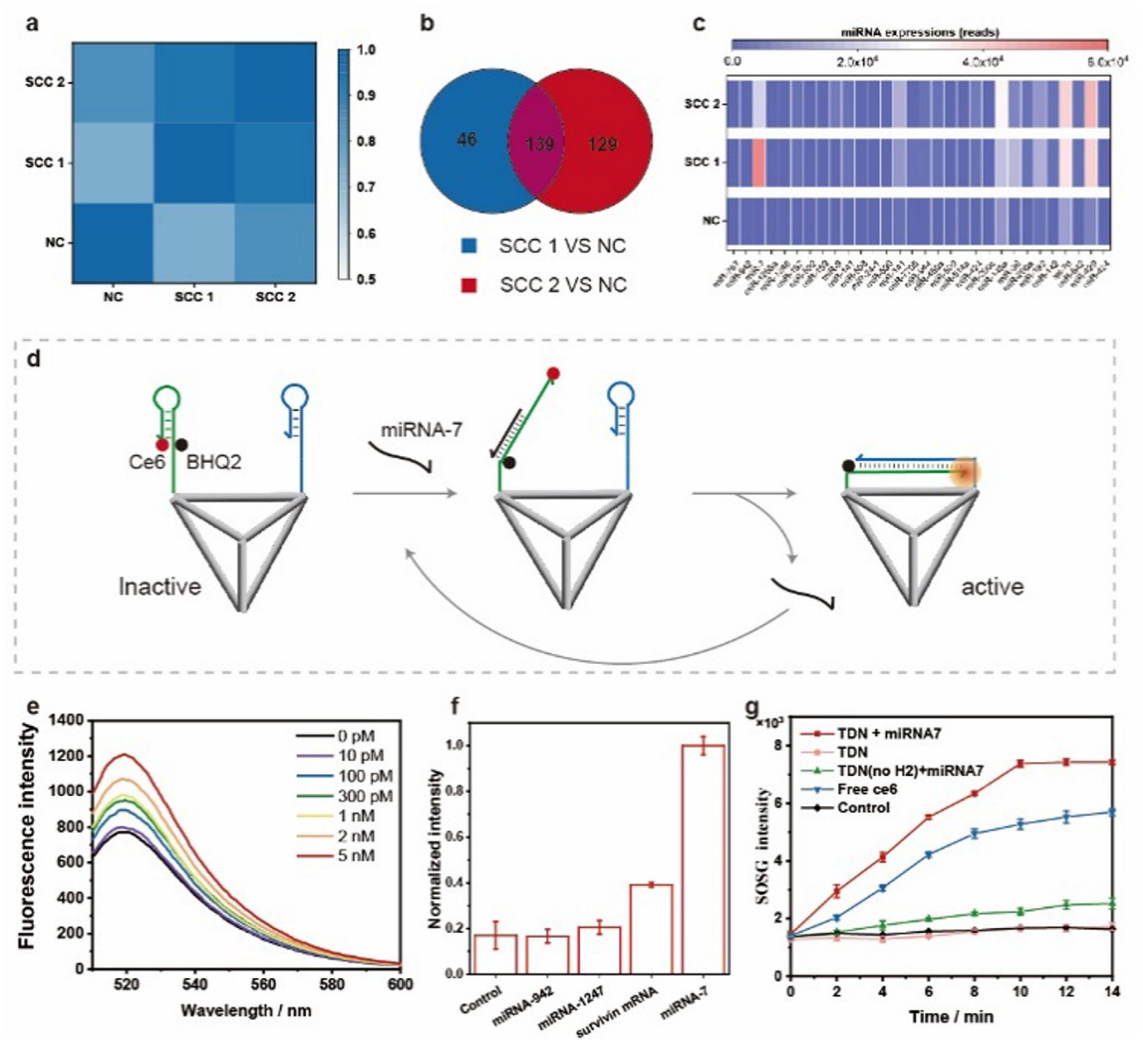
The screen of cSCC‐specific miRNA biomarkers and validation of miRNA‐responsive singlet oxygen amplification. The correlation heatmap a) and DEGs b) of the RNA sequencing results from two tumor tissue samples and one healthy human tissue. c) The top 25 up‐regulated miRNA expression profiles from 139 DEGs. d) The working principle of miRNA7‐mediated HCA reaction. e) Fluorescence spectroscopy of FAM‐labeled TDN that was monitored at an excitation wavelength of 488 nm, within a series of miRNA‐7 concentrations. f) Selective recognition ability of TDN toward different RNA species. g) The singlet oxygen production of different groups under light irradiation and H_2_O_2_ supply, moniterd by using SOSG probes.

After successfully constructing TDN, it was designed to target the cSCC tumor. To achieve a tumor‐specific miRNA response, miRNA markers that are closely related to cSCC need to be identified. At first, two tumor tissue samples from patients and one tissue sample from a healthy person were obtained for RNA sequencing analysis. According to the correlation heatmap of the sequencing results, the two tumor tissue samples were in good agreement and significantly different from those of healthy human tissues (**Figure** **2**a). Both tumor tissues contained 139 differentially expressed genes (DEGs), compared to that of control samples (Figure 2b). Next, 25 up‐regulated miRNAs were successfully identified, and their stable expression levels in the cSCC group were several times higher than those in the healthy group (Figure S5, Supporting Information). From the upregulated miRNA expression profiles, miRNA‐7 was selected as a candidate miRNA target associated with cSCC (Figure 2c). Furthermore, to verify that miRNA‐7 was indeed closely related to cSCC, we subsequently performed bioinformatics analysis using the public TCGA database. The expression of miRNA‐7 was significantly higher in skin tumors than in normal tissues (Figure S6, Supporting Information). Subsequent Kyoto Encyclopedia of Genes and Genomes (KEGG) enrichment analysis revealed that the signaling pathways of miRNA‐7 were mainly associated with autophagy, endocytosis, ErbB signaling pathway, etc (Figure S7, Supporting Information). Abnormal signaling of ErbB family members plays an important role in the occurrence and development of various malignant tumors.^[^ ^35^ ^]^ Therefore, miRNA‐7 was chosen as the target for TDN to specifically respond to cSCC.

When miRNA‐7 was present, the hairpin H1 on TDN was first reacted to activate the PS. Subsequently, the newly exposed toehold domain on H1 underwent a competitive strand displacement reaction with hairpin H2, replacing and releasing miRNA‐7 (Figure 2d). Therefore, a small amount of miRNA‐7 can activate a large number of PSs through catalytic amplification. To verify the effectiveness of this process, we first replaced the photosensitizer on hairpin H1 with a FAM fluorophore. Using a DNA target identical to the miRNA‐7 sequence, the fluorescence recovery of TDN was tested with a range of target concentrations (Figure 2e). On the other hand, Dox fluorescence recovery was also recorded in the presence of miRNA‐7. The increasing fluorescence kinetic over time supported the controlled release of Dox (Figure S8, Supporting Information). As the target concentration increased, the fluorescence intensity increased gradually. The recognition sensitivity of TDN was estimated at 0.7 pm (Figure S9, Supporting Information), which can effectively respond to low‐abundance miRNAs in tumor cells. In the other hand, the selectivity of the TDN toward RNA molecules was investigated. The results showed that TDN had good selectivity (Figure 2f). The peroxidase and catalase activity of DNA nanomachines after embedding hemin molecules had also been validated. In the presence of H_2_O_2_, TDN can effectively oxidize TMB to a blue complex (Figure S10, Supporting Information) and cleave H_2_O_2_ to produce oxygen molecules (Figure S11, Supporting Information).

Subsequently, in vitro operation of TDN modified with Ce6 molecules was tested. When a target was present, the fluorescence peak of Ce6 at 664 nm was restored (Figure S12, Supporting Information). Under light irradiation, ce6 molecules react with the cellular oxygen to produce singlet oxygen molecules. To demonstrate the ROS amplification effect of DNA nanomachines in response to miRNA targets, the production of singlet oxygen under light and in presence of H_2_O_2_ conditions was detected by singlet oxygen sensor green (SOSG) fluorescent probes. Compared to the control groups without hairpin H2 or target miRNA‐7, the production of singlet oxygen by TDN was significantly high (Figure 2g), even higher than the production by free Ce6 at the same concentration (500 nm). It assessed the ability of TDN to achieve HCA‐based singlet oxygen amplification in addition to the advantageous miRNA‐7 responsive activation that prevented the phototoxicity of Ce6.

### 2.3 Aptamer‐Mediated Cellular Uptake and Selective Activation

**Figure 3:**
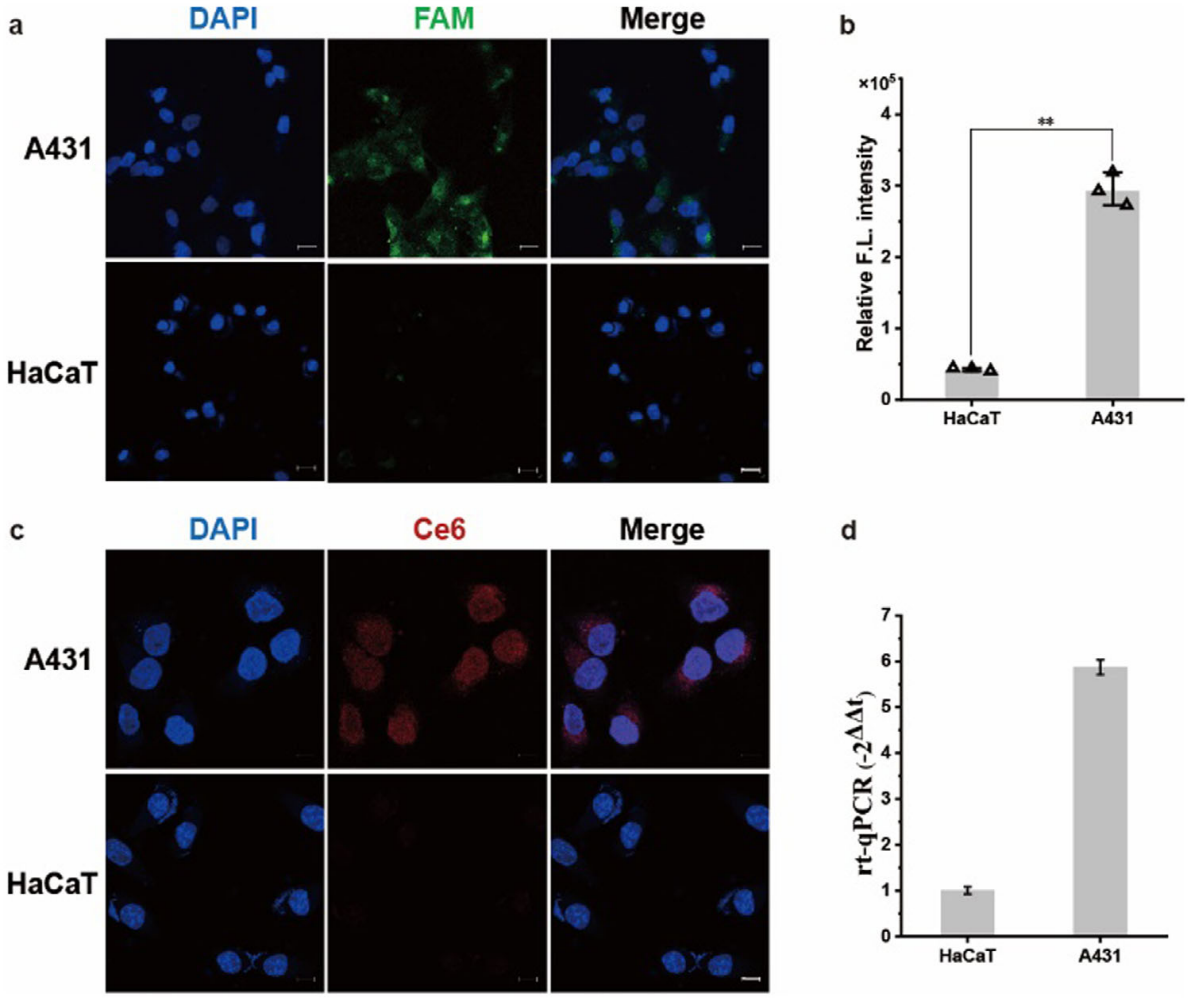
Cellular uptake and intracellular miRNA‐responsive activation of TDN. a) CLSM images of FAM‐labeled TDN to study AS1411 aptamer‐mediated cell uptake and miRNA‐responsive green fluorescence recovery. Scale bar is 20 µm. b) The relative fluorescence intensity of FAM‐labeled TDN in A431 and HaCaT cells. c) CLSM images of Ce6‐labeled TDN in tumor cells and normal cells. Scale bar is 10 µm. d) RT‐qPCR analysis of intracellular miRNA‐7 expression level in A431 and HaCaT cells.

Next, aptamer‐mediated cellular uptake and intracellular selective activation were studied. AS1411 aptamer is an artificially selected oligonucleotide sequence that can specifically bind to nucleolin protein, which is highly expressed on the membrane of various tumor cells including cSCC.^[^ ^36^ ^]^ First, we used the CCK‐8 experiment to verify that the DNA tetrahedron nanostructures had good biocompatibility (Figure S13, Supporting Information) and nuclease stability against enzyme attack (Figure S14, Supporting Information). Human epidermal cancer cell line A431 and human normal cutaneous keratinocyte line HaCaT were respectively selected as positive and negative cells for the study. Aptamer‐mediated uptake and miRNA‐7‐selective fluorescence activation was visualized via confocal laser scanning microscopy (CLSM), by using FAM‐labeled H1 to construct TDN. The time‐varying fluorescence recovery in both cells was investigated (Figure S15, Supporting Information). The fluorescence brightness of TDN in the cytoplasm of A431 was progressively enhanced, suggesting that TDN can target tumor cells and lead to fluorescence recovery after reacting with miRNA‐7 in the cytoplasm. In contrast, little fluorescence signal was observed in HaCaT cells. At ≈16 h, the fluorescence brightness reached a stable plateau (**Figure** **3**a). Relative intensity analysis of the images using ImageJ showed that the fluorescence intensity in A431 cells was about five times higher than that of HaCaT cells (Figure 3b).

Further, FAM‐labeled DNA was replaced with Ce6‐labled DNA while constructing PS‐labled TDN. The fluorescence recovery of Ce6 in two cells was verified under the same conditions. The results remained in line with our expected judgments. To confirm that the fluorescence activation of ce6 in different cells correlates with the intracellular miRNA‐7 expression level, we performed RT‐qPCR analysis (Figure 3d). The results revealed that the level of miRNA‐7 in A431 cells was about six times higher than the level in HaCaT cells. Basically, the result was consistent with the CLSM results. Therefore, it was demonstrated that TDN can specifically target to tumor cells and selectively activate PSs, through sequential recognition of nucleolin and miRNA‐7.

### 2.4 In Vitro Evaluation of Photodynamic Therapeutic Efficacy

**Figure 4:**
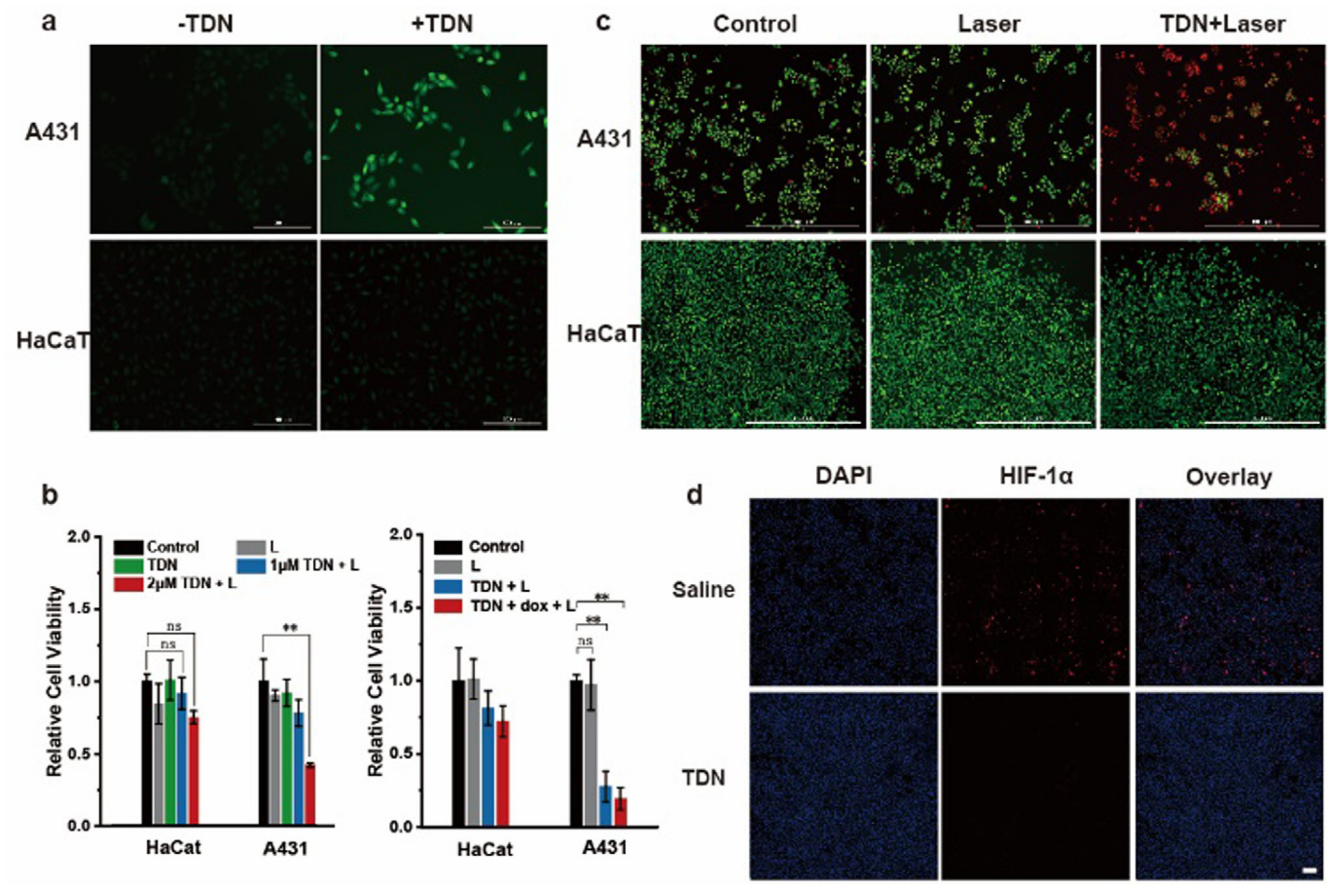
The evaluation of ROS production and in vitro photodynamic therapeutic efficacy. a) Fluorescence imaging of ROS by DCFH‐DA staining in A431 cells and HaCaT cells with or without the treatment of TDN. The scale bar is 200 µm. b) CCK‐8 assays of tumor cells and normal cells after different treatment and light irradiation. c) Calcein AM/PI double stain of A431 cells and HaCaT cells after various treatments. Scale bar is 1000 µm. d) Immunostaining imaging results of intracellular HIF‐1α. The scale bar is 100 µm.

It had been demonstrated that TDN had an effective intracellular transfection ability of PSs with high selectivity toward tumor cells and responsive activation by miRNA‐7, we proceeded to evaluate photodynamic therapy efficacy in cells. First, the intracellular ROS production under laser irradiation was evaluated, by using 2′, 7′‐dichlorofluorescin diacetate (DCFH‐DA) as an indicator. DCFH was oxidized to DCF by ROS and emitted green fluorescence. In the absence of TDN, only a faint fluorescence intensity can be observed in both tumor cells and normal cells after irradiation. After a period of co‐incubation with TDN, bright green fluorescence was detected in tumor cells A431, indicating that a large amount of ROS was produced within the cells (**Figure** **4**a). Green fluorescence was still hardly observed in normal cells HaCaT, because it was unable to activate PSs due to the lack of miRNA‐7 in the cytoplasm, which prevented sufficient ROS production.

High levels of ROS can cause damage to cellular structures and genetic material and ultimately induce cell death. The cell killing effect and proliferation inhibition after TDN administration were investigated by the CCK‐8 method. The group with laser irradiation after TDN administration was the experimental group. Laser only and TDN without laser irradiation groups were used as control. As shown in Figure 4b left, cell viability in the control group showed little to no change within 24 h after treatment. After 1 µm TDN administration and light irradiation, a tiny killing effect was observed within tumor cells. This may be attributed to the fact that the effective dose is too low to kill the cells. When the concentration was increased to 2 µm, the viability of A431 cells decreased substantially to reach less than 50%, while the HaCaT showed a maintained high survival rate. The result emphasized that a sufficient amount of TDN would specifically be activated and amplified by miRNA in tumor cells, leading to PDT‐mediated cell death. To enhance the anti‐tumor effect, Dox was embedded in hairpin H1. The cells survival rate of tumor cells was further reduced while normal cells still had little change (Figure 4b, right), achieving an effective synergistic therapy. To further analyze the induced apoptosis, Calcein AM/PI (propidium iodide) double staining was used to determine the ratio of living cells versus dead cells. For HaCaT cells, dead cells were barely observed after TDN administration and light irradiation. In A431 cells, the red fluorescence brightness increased greatly after TDN administration and illumination, indicating the apoptosis of a large number of tumor cells (Figure 4c). In addition, PDT efficacy is conditioned by the abundance of in situ oxygen molecules. However, tumors are characterized by a highly hypoxic microenvironment that hinders PDT efficiency.^[^ ^37^ ^]^ TDN was designed to combine with hemin, for the sake of the improvement of tumor oxygenation and the intracellular photodynamic effect. To verify the PDT mechanism with oxygen self‐supply, immunostaining assays (Figure 4d) and western blot assay (Figure S16, Supporting Information) of hypoxia inducible factor‐1α (HIF‐1α) were performed. Both results showed a significant decrease of HIF‐1α after TDN treatment, proving that the relief of intracellular hypoxia by AS1411‐hemin catalase‐like complex promoted the low expression of HIF‐1α. Interestingly, the HIF‐1α band treated by AS1411‐hemin alone had little change compared to the control group in the western blot result. This may be due to the difficulty of single strand in entering cells to play function, as well as its quick degradation. Therefore, it was demonstrated that TDN combined with hemin and Dox molecules exhibited a high level of ROS production, and selective phototoxicity and chemotherapy effects on tumor cells.

### 2.5 In Vivo Antitumor Efficiency and Biosafety Evaluation

**Figure 5:**
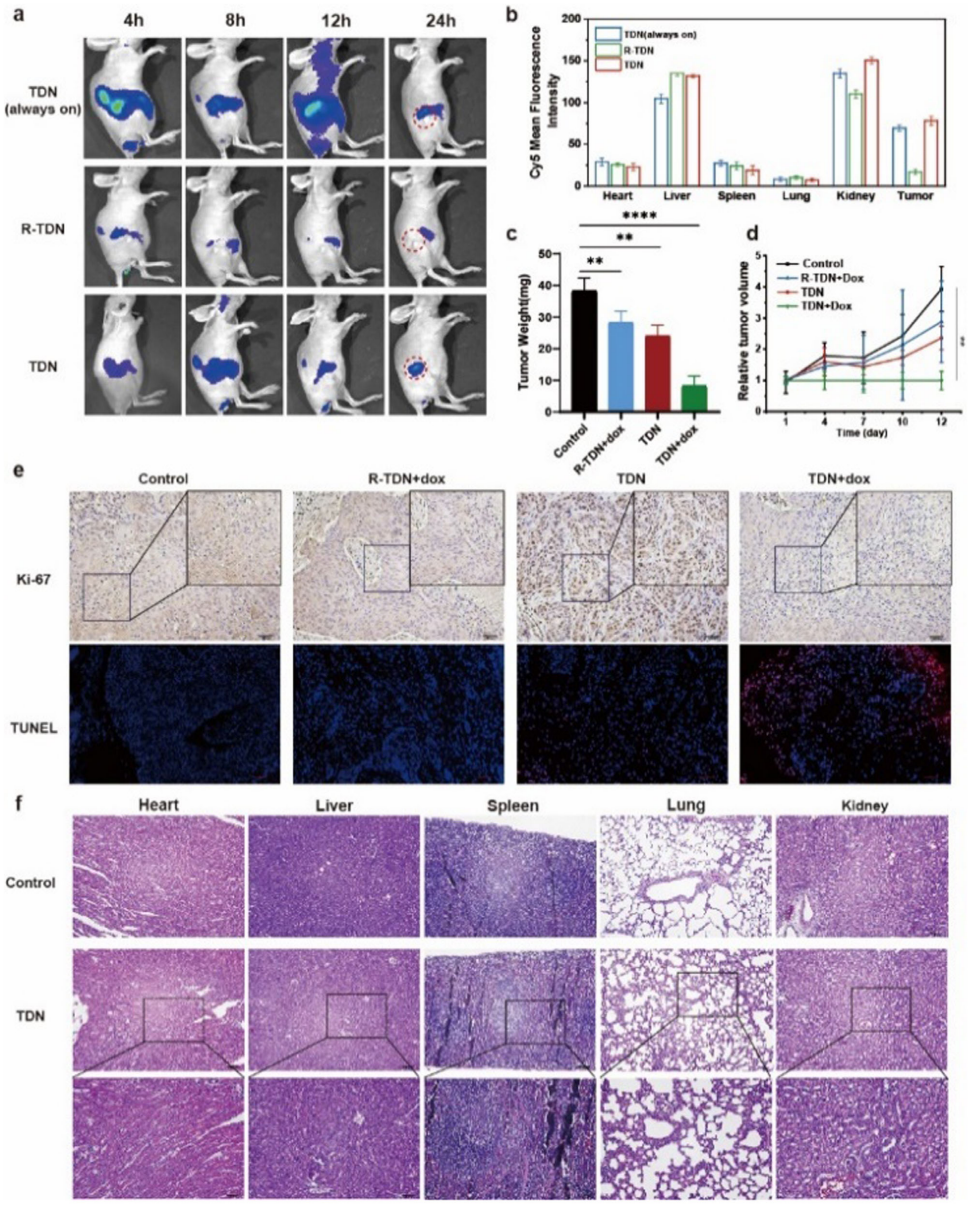
Study on in vivo anti‐tumor effect and biosafety. a) The biodistribution with different treatments by Cy5.5 fluorescence images of mice taken at 4, 8, 12, and 24 h post intravenous injection. b) Quantitative fluorescence intensity of major organs after 24 h administration. c) Tumor weight of the mice in corresponding groups. d) Relative tumor growth curves of the mice in corresponding groups. e) Immunofluorescence staining of Ki‐67 and TUNEL in tumor tissue. Scale bar is 100 µm. f) H&E immunohistology images of tumors in the phototoxicity experiments.

After the assessment of in vitro singlet oxygen amplification and intracellular PDT efficacy, in vivo anti‐tumor application of TDN was further explored. A431 tumor‐bearing BALB/c nude mouse model was successfully constructed by subcutaneous injection of A431 cells. First, Cy5.5 dye was used to replace Ce6, to study the biodistribution of TDN and its in vivo activation. Positive control was set up by removing the quencher from the TDN, so that the Cy5.5 fluorescence was always on without the need of miRNA activation. The sustained high signal was detected in the positive group during the observation time, and there was still significant accumulation at the tumor site at 24 h. Compared to the positive group, TDN group showed a gradually concentrated distribution of the fluorescence signal at the tumor site due to the specific activation of miRNA‐7 (**Figure** **5**a). However, when the miRNA‐7‐recognizing H1 was replaced by a random sequence, the result showed very weak signal, confirming miRNA7‐selective activation of TDN group. Moreover, ex vivo imaging of the harvested organs and tumors after 24 h also showed that in addition to being metabolized and eliminated by the liver and kidney, high fluorescence was observed in tumor sites (Figure 5b; Figure S17, Supporting Information). It suggested that TDN could actively target tumor tissue after intravenous administration and is selectively activated by miRNA‐7.

Next, the anti‐tumor effect of TDN in combination with Dox and PSs was evaluated. When the tumor volume grew to a size of ≈50 mm^3^, different groups were intravenously injected every 3 days during a 12 day administration. Light irradiation (630 nm, 200 mW cm^−2^) was performed after 24 h post‐injection, according to the biodistribution study. The anti‐tumor effect was observed through monitoring tumor volumes and body weight, as well as the photographs of harvested tumors taken after euthanasia on day 12. Compared to the saline‐treated group, both Ce6‐labeled TDN and R‐TDN with Dox can promote minor tumor ablation. TDN combined with PS and Dox together showed a significant regression of the tumors (Figure 5c,d; Figure S18, Supporting Information), demonstrating a synergistic anti‐tumor effect. Furthermore, the tumor tissues obtained from each group were analyzed by Ki‐67 and TUNEL immunostaining (Figure 5e; Figure S19, S20, Supporting Information). The results showed that the proliferative activity of tumor cells in the combined treatment group was effectively inhibited, and a large number of tumor cells showed apoptosis. The immunohistochemical analysis was consistent with the previous statements. Overall, in vivo experiments demonstrated that TDN had a high anti‐tumor effect on cSCC through a combination of photodynamic therapy and chemotherapy.

Throughout the administration, the body weight was monitored and presented minor changes in all groups (Figure S21, Supporting Information). There were no obvious pathological structural changes in the major organs including heart, liver, spleen, lungs, and kidneys, after administration (Figure S22, Supporting Information), demonstrating good in vivo biosafety of TDN. To further evaluate whether TDN can reduce off‐target phototoxicity, a high dosage and long‐time light irradiation were used to mimic accidental light exposure. Three groups including saline, TDN without quencher, and TDN, were set. The hematoxylin and eosin (H&E) staining showed that there were no obvious inflammatory infiltrates or pathological structural changes in the heart, liver, spleen, lungs, and kidneys (Figure 5f; Figure S23, Supporting Information). In another phototoxicity experiment, PSs transfected by commercial liposome was administered as an experimental group, with an equivalent dosage of PSs. As a result, this group showed less anti‐tumor effect, but more phototoxicity‐induced inflammatory reaction (Figure S24, Supporting Information), compared to the TDN group. It witnessed that TDN could effectively reduce the off‐target phototoxicity and would not cause abnormal inflammatory lesions and damage in the body, due to the two features of tumor‐specific targeting and miRNA‐responsive PDT activation.

## 3 Conclusion 

In conclusion, we developed an intelligent DNA nanomachine based on tetrahedral DNA framework to achieve precise and high‐efficient treatment of cSCC tumor. In this design, tumor‐specific therapy comes from two aspects. The first is the specific recognition of nucleolin proteins that were highly expressed on the membrane of tumor cells, through the AS1411 aptamer carried by TDN. Secondly, the PSs of transfected TDN can only be activated by recognizing specific miRNA‐7 in the cytoplasm. Based on the tumor‐targeting capability, photodynamic and chemotherapy are combined to achieve precise and high‐efficient treatment of cSCC. Importantly, HCA reaction was properly designed in TDN to amplify the activated dose of PSs and realize controlled release of Dox molecules, ensuring the mass generation of singlet oxygen and the continuous killing effect on of tumor tissues.

Through RNA sequencing of clinical tissue samples and bioinformatics analysis, a cSCC tumor‐related miRNA molecule was screened out. MiRNA‐7 is highly expressed in tumor cells, but hardly expressed in normal cells. This is significant guarantee to establish miRNA‐responsive singlet oxygen amplification and tumor precision therapy. A series of in vitro and in vivo experimental results were performed to prove the success of this strategy. It provides a feasible and valuable non‐invasive weapon for the precision treatment of cSCC as well as other skin‐related tumors. More importantly, this strategy not only improves the therapy efficacy, but also solves some problems in the clinical application of PDT, including off‐target phototoxicity and hypoxic environment‐induced low efficacy. We believe that this strategy will be a strong candidate for anti‐tumor therapy of cSCC and have great potential in the clinical treatment of other skin tumors.

## Conflict of Interest

The authors declare no conflict of interest.

## Supporting information



Supporting Information

## Data Availability

The data that support the findings of this study are available from the corresponding author upon reasonable request.
