# Supplementary material for: Targeted DNA Nanomachine Enables Specific miRNA‐Responsive Singlet Oxygen Amplification for Precise Cutaneous Squamous Cancer Therapy
Source: Adv Sci (Weinh). 2025 Mar 21;12(19):2415296. doi: 10.1002/advs.202415296 (PMC12097105; doi:10.1002/advs.202415296)
Supplement: Supplementary file 1 — Supporting Information [file ADVS-12-2415296-s001.pdf]

ADVANCED SCIENCE
Open Access

Supporting Information

for *Adv. Sci.*, DOI 10.1002/advs.202415296

Targeted DNA Nanomachine Enables Specific miRNA-Responsive Singlet Oxygen Amplification for Precise Cutaneous Squamous Cancer Therapy

Hanane Aliouat, Detian Zhang, Lanyuan Peng, Jiaxin Huang, Hongshi Cheng, Jiaojiao Zhu, Xiang Chen, Nuli Xie, Wenhu Zhou* and Shuang Zhao**

Supporting Information

Targeted DNA nanomachine enables specific miRNA-responsive singlet oxygen amplification for precise cutaneous squamous cancer therapy

Hanane Aliouat[†1], Detian Zhang[†2,3], Lanyuan Peng[2], Jiaxin Huang[1], Hongshi Cheng[1], Jiaojiao Zhu[1], Xiang Chen[2], Nuli Xie[1]*, Wenhu Zhou[1]*, Shuang Zhao[2]*

[1]Xiangya School of Pharmaceutical Sciences, Central South University, Changsha, Hunan 410013, China. Furong Laboratory, Changsha, Hunan 410008, China.

[2]Department of Dermatology, Hunan Engineering Research Center of Skin Health and Disease, Hunan Key Laboratory of Skin Cancer and Psoriasis, Xiangya Hospital, Central South University, Changsha, Hunan 410008, China. Furong Laboratory, Changsha, Hunan 410008, China.

[3]Hangzhou Innovation Institute, Beihang University, Hangzhou, Zhejiang 310053, China.

*To whom correspondence should be addressed:
shuangxy@csu.edu.cn; nulixie@csu.edu.cn; zhouwenhuyaoji@163.com

Contents

S1. Supplementary Methods Page 2-7
S2. Supplementary Figures Page 8-19
S3. DNA sequences Page 20

S1 Supplementary Methods

Materials

The DNA strands were bought and purified from Sangon Biotech Co., Ltd (Shanghai, China). Chlorin e6 (ce6)-modified DNA was obtained and purified by Takara Bio Inc. (Beijing, China). The DNA sequences were listed in **Table S1**. RNA sequencing was performed by BGI company (Shenzhen). The 3,3',5,5' tetramethylbenzidine (TMB) was produced by Shanghai Aladdin Biochemical Technology Co. Ltd. Hemin was obtained from Macklin Co., Ltd (Shanghai, China). Potassium chloride (KCl), magnesium chloride ($MgCl_2$) and Sodium chloride (NaCl) were issued from Sinopharm Chemical Reagent Co., Ltd. (Shanghai, China). PBS buffer was from ServiceBio Co., Ltd (Wuhan, China). Calcein AM/PI double stain kit, Singlet oxygen sensor green probe (SOSG) and 2',7'-dichlorofluorescein diacetate (DCFH-DA) were obtained from Shanghai Maokang biotechnology Co., Ltd. Hoechest was produced by Solarbio life science, Beijing, China and the 4% paraformaldehyde was from AiFang biological, China. The electrophoresis precast gel was from ACE Biotechnology. Doxorubicin was purchased from Macklin Biochemical (Shanghai, China). All reagents used in the cellular experiment were purchased from Gibco Life Technologies (Grand Island, NY, USA). All reagents are used in analytical purity guaranteed by the companies.

Preparation and Characterization of TDN

All DNA strands were appropriately dissolved in double distilled water and quantified by UV spectrophotometer at 260 nm wavelength. According to the reported protocol, four strands (S1, S2, S3, S4) were mixed in equimolar to assemble tetrahedral DNA nanostructure, by heating to 95°C for 5 min, then cooling to room temperature. Strand S3 was extended at 3' end with AS1411 aptamer sequence. Two hairpin DNA molecules (ce6-labeled H1 and H2) were pinned to the unhybridized domain of strand S1 and S2 respectively, to form tetrahedral DNA nanomachine (TDN). In addition, Tris-Mg buffer was replaced by PBS buffer (12.5 mM $MgCl_2$) when preparing TDN for animal experiments. For inactivation of ce6, BHQ2 group was modified at the 5' end of strand S1 to quench the ce6 molecule in proximity. For Hemin loading, TDN was incubated with Hemin dissolved in DMSO solution at a molar ratio of 1:2 for 6 h in the dark environment. For chemotherapeutic drug loading, the H1 was incubated with Doxorubicin (Dox) at a ratio of 1:5 by stirring at 200 rpm for 3 hours. Excess Dox and hemin molecules can be removed through ultrafiltration centrifugation with 3 kDa MWCO tubes.

To characterize TDN, 12% native polyacrylamide gel electrophoresis (PAGE) was performed. Different samples including S1, S1+S2, S1+S2+S3, DNA tetrahedron

(S1+S2+S3+S4), TDN (S1+ S2+ S3+ S4 +H1 +H2), H1 and H2, were prepared as requested, followed by mixing 10 μL DNA with 2 μL 6× loading buffer. The gel was run on electrophoresis device (Bio-Rad, USA) at 100 V for 2 h. The gels were stained with 1× GelRed dye before scanning by a ChemiDoc XRS+ imager (Bio-Rad, USA). In addition, atomic force microscopy (AFM) and dynamic light scattering (DLS) were also used to study TDN. For AFM experiment, 5 μL samples were pipetted onto freshly prepared mica surface. After 5 min incubation, the surface was washed three times by buffers, then imaged by a MultiMode Nanoscope VIII system (Bruker). For DLS experiment, sample was filtered using a 0.22 μm Millipore nylon syringe filter before testing. 100 μL sample was placed in a cuvette and measured three times by a Zetasizer Nano ZS instrument (Malvern, USA).

To test the effectiveness of Hemin incorporation, a UV-Vis spectrophotometry was performed after the incubation of strand S3 and TDN with Hemin at a ratio of 1:2 at 25 °C for 6 h followed by ultrafiltration centrifugation to discard the excess Hemin. Hemin alone that has a peak at 400 nm was used as control.

Fluorescence analysis

The fluorescence experiments were determined using F-2700 fluorescence spectrometer (HITACHI, Japan). To investigate the response ability of TDN to miRNA target, FAM-labeled H1 was used to replace the ce6-labeled H1. 100 nM of TDN was incubated with a series of gradual concentrations (0 pM, 10 pM, 100 pM, 300 pM, 1 nM, 2 nM, 5 nM,) of miRNA target at 37 °C for 2 h. Fluorescence emission spectrum of FAM (Ex/m =488 nm /520 nm) was recorded. The fluorescence intensity without target was recorded as the background. A linear curve was plotted and the limit of detection was determined by the 3σ/slope formulation. In the selectivity studies, three non-specific targets including survivin mRNA, miRNA-942 and miRNA-1247-3 were investigated. The concentrations were kept in same with that of the experiment group. The fluorescence activation of ce6 (Ex/Em =405 nm /664 nm) was also confirmed by using 500 pM miRNA-7 target.

To test the effectiveness of Dox loading, the fluorescence spectrum of Dox was studied. Red fluorescence of free Dox can be quenched when intercalated into DNA duplex domain. Free Dox and the supernatant solution after intercalation were detected under an excitation wavelength at 488 nm, and collected in the range of 560-700 nm.

Nuclease stability

TDN samples at a concentration of 5 μM were mixed with 0.5 U of DNase I at 37 °C to investigate the structure stability at several time points (0 h, 1 h, 2 h, 4 h, 6 h, 16 h, 24 h). After terminating reaction, all samples were assessed by running 12% native

PAGE at 100 V for 1 h.

Catalase-like activity and singlet oxygen (O_2^1) generation

The complex of G-rich domain and Hemin has catalase-like ability to produce oxygen in presence of H_2O_2. 1 μM of TDN-hemin complex was added to 100mM of H_2O_2 in a total volume of 1 mL. Oxygen levels were measured during 10 min by the portable dissolved oxygen meter (JPBJ-609L, INESA Scientific Instrument Co., Ltd., China). Also, the peroxidase activity was assessed through TMB reaction in presence of H_2O_2. Briefly TMB (0.5 mM), H_2O_2 (2 mM) and TDN-hemin complex (1 μM) were mixed in a total volume of 1 mL. The absorbance of oxidized TMB was assessed by UV spectrophotometry at wavelength of 660 nm.

The O_2^1 production was measured using the singlet oxygen sensor green (SOSG) probe. The fluorescence intensity (Ex/Em = 504 nm /525 nm) of different samples which had same concentration of ce6 were measured at different time points (0-15 min), under laser irradiation (630 nm, 0.1 W/cm^2). Besides ce6-labeled TDN sample, TDN without hairpin H2 and free ce6 were set as control group. All the samples are mixed with SOSG and H_2O_2 (100 mM) before measurement.

Cell culture

Human skin carcinoma cell lines A431 and human immortalized keratinocyte cell lines HaCaT were used as cancer cell and normal cell in this project, respectively. Cells were grown in DMEM medium with 10% fetal bovine serum (FBS, Gibco) and 1% penicillin-streptomycin in 5% CO^2 atmosphere at 37 °C. For confocal imaging experiments, cells were firstly cultured on 35-mm dishes or 6-well plates at least 24 h, then washed by PBS buffer (pH 7.4) three times before subsequent steps.

Fluorescence imaging

A431 cells and HaCaT cells were incubated with 200 nM FAM-labeled or ce6-labeled TDN (with quencher) in serum-free medium at 37 °C dark environment for varied time (0 h, 4 h, 8 h, 16 h) or 16 h to study cellular uptake. The cells were fixed with paraformaldehyde (4%) for 10 min, and washed twice with PBS. Subsequently, the cells were treated by nucleus-staining Hochest dye for 10 min, and washed twice with PBS. Finally, each dish was imaged by confocal laser scanning microscope (CLSM, LSM780 NLO, Zeiss, Oberkochen, Germany), under Ex/m wavelengths of FAM (488 nm /520 nm) or ce6 (405 nm /670 nm).

To investigate the capability of intracellular O_2^1 production, A431 cells and HaCaT cells were seeded in the 96 wells plate at a density of 8×10^3/ well, then treated with TDN and laser irradiation. The samples without addition of TDN were used as control.

After washing twice with PBS, DCFH-DA was added at 37 °C for 0.5 h. The intracellular levels of O_2^1 were visualized by fluorescence microscopy (Cytation 5, Biotek, USA).

RT-qPCR of intracellular miRNA-7

The miRNA-7 expression level in A431 and HaCaT cells was verified by qRT-PCR that was performed by commercial company (Wuhan Servicebio technology Co. Ltd). Briefly, total cellular RNA was extracted using Trizol reagent. Then, the reverse transcription (RT) reaction was performed, followed by qPCR analysis on a LightCycler480 Software Setup (Roche). All data were evaluated with respect to the miRNA expression by normalizing to the expression of U6 and using the $2^{-\Delta\Delta Ct}$ method. The primers (from 5' to 3') used in this experiment were listed below.

miRNA-7 forward: ACACTCCAGCTGGGTGGAAGACTAGTGATTTT

miRNA-7 reverse: CTCAACTGGTGTCGTGGAGTCGGCAATTCAGTTGA GAACAACAA

U6 forward: CTCGCTTCGGCAGCACA

U6 reverse: AACGCTTCACGAATTTGCGT

***In vitro* cytotoxicity analysis**

CCK-8 experiments were conducted to study the cytotoxicity of TDN. A431 and HaCaT cells were seeded in 96 wells plates with a density of 8×10^3/well and incubated for 24 h. TDN was added at a concentration of 1μM and 2 μM and incubated for 8 h, then cells were irradiated by laser for 10 min. The medium was discarded after another 12 h culture. The cells were treated with CCK-8 reagent for 2 h. Finally, the absorbance at 450 nm was read by microplate (Tecan, Switzerland) to calculate the cell viability. In another experiment, a comparison between TDN and Dox-intercalated TDN was conducted to assess the synergistic killing effect.

Furthermore, Calcein AM/PI staining for live/dead cells was also performed to study the apoptosis resulted from the O_2^1 cytotoxicity. Cells were cultured to 80% in a 6-well plate. The treatment of TDN and laser irradiation was same to the described above. Latterly, it was visualized by fluorescence microscopy (Cytation 5, Biotek, USA), with red fluorescence showing dead cells and green fluorescence showing live cells.

***In vivo* biodistribution analysis**

Female Balb/c nude mice (6-8 weeks old) were procured from Tianqin Biotech. Co., Ltd. (Changsha, China). All experiments with mice are conducted in accordance with animal ethical standards and guidelines. Additionally, the experiment protocols were reviewed and approved by Medical Ethics Committee of Xiangya Hospital Central

South University (202308636) prior to the research. A431 tumor-bearing mouse models were established by injecting A431 cells (1× 10^6 cells, 0.2 mL) into Balb/c nude mice. Once tumor sizes reached around ~100 mm^3, the mice were injected intravenously with TDN (without BHQ2, always on), R-TDN (random H1), and TDN, at an equivalent dose of 0.5 nmol of Cy5.5. The mice were imaged by using an in vivo imaging system at 4, 8, 12, and 24 h post-injection. After 24-h administration, the tumor and main organs (heart, liver, spleen, lung, and kidney) were harvested and imaged.

In vivo antitumor effect

To evaluate the in vivo therapeutic efficacy, A431 tumor-bearing mouse models were established according to the protocol described above. When tumor sizes reached around ~100 mm^3, the mice were randomly divided into four groups and injected intravenously with different formulas (an equivalent dose of 1.0 nmol of ce6), including saline, R-TDN with Dox, TDN, and TDN with Dox. The drugs were administrated every 3 days, four times in total. After 24 h post-injection, mice were irradiated at tumor sites for 10 min (630 nm, 0.2 W/cm^2). Tumor volumes and body weight were monitored daily throughout a 12-day period. Tumor volume was calculated using the formula $V = L \times W^2/2$, where L represented the tumor length and W represented the tumor width. Relative tumor volume was denoted as changes relative to the initial volume before treatment. The mice were treated by humane euthanasia on day 12. The tumor and main organs (heart, liver, spleen, lung, and kidney) were harvested for immunohistology analysis of TUNEL, Ki-67, and H&E staining.

In vivo phototoxicity analysis

The tumor-bearing mice were randomly divided into 3 groups and treated with saline, TDN (always on), and TDN, respectively (an equivalent dose of 2.0 nmol of ce6). Subsequently, the mice were subject to photoirradiation for 1 h (630 nm, 50 mW/cm^2) and monitored for another 24 h. To evaluate the safety, the blood was collected to analyze the levels of aspartate aminotransferase (AST), alanine aminotransferase (ALT), blood urea nitrogen (BUN), and creatinine (CRE). The main organs were harvested for H&E staining.

In another phototoxicity experiment, the tumor-bearing mice were randomly divided into 3 groups and treated with saline, Ce6 transfected by liposome, and TDN, respectively (an equivalent dose of 2.0 nmol of ce6). The subsequent examination and image analysis are same as the above.

Statistical analysis

Statistical analyses were performed using Origin scientific software. Image

analysis was performed using ImageJ software. All results were calculated as mean value ± standard deviation. Single t test and one-way analysis of variance were used to assess the significant differences. The significance was defined as follows: *$P < 0.05$, **$P < 0.01$, ***$P < 0.001$, and ****$P < 0.0001$.

S2 Supplementary Figures

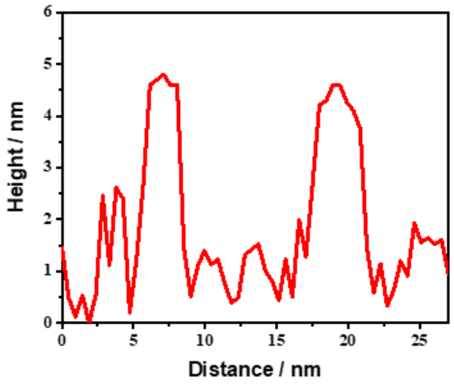

Figure S1. Height sectional view of TDN from the AFM image. It indicated that TDN had a height of about 5 nm, which was in accordance with the theoretical value.

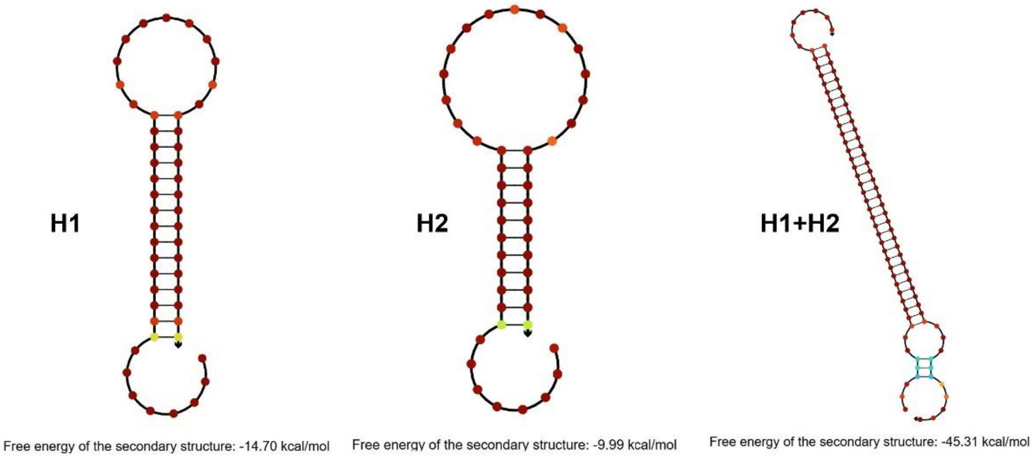

Figure S2. Nupack simulations to analyze the secondary structure and free energy of DNA hairpin molecules used in TDN.

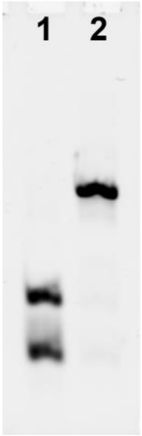

Figure S3. PAGE analysis of catalytic hairpin assembly (CHA) reaction in the absence or presence of DNA target. Lane 1: H1 + H2; Lane 2: H1 + H2 + target.

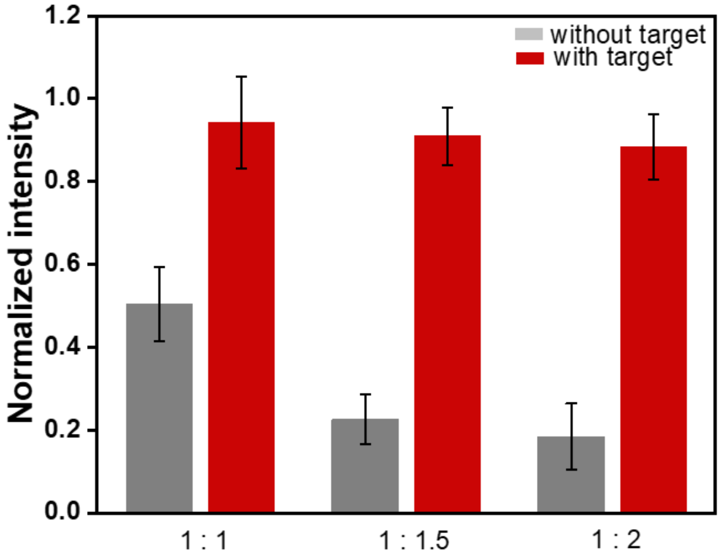

Figure S4. Optimization of the ratio of hairpin DNA and DNA tetrahedral nanostructures, to guarantee efficient inhibition of ce6 activattion in the absence of target.

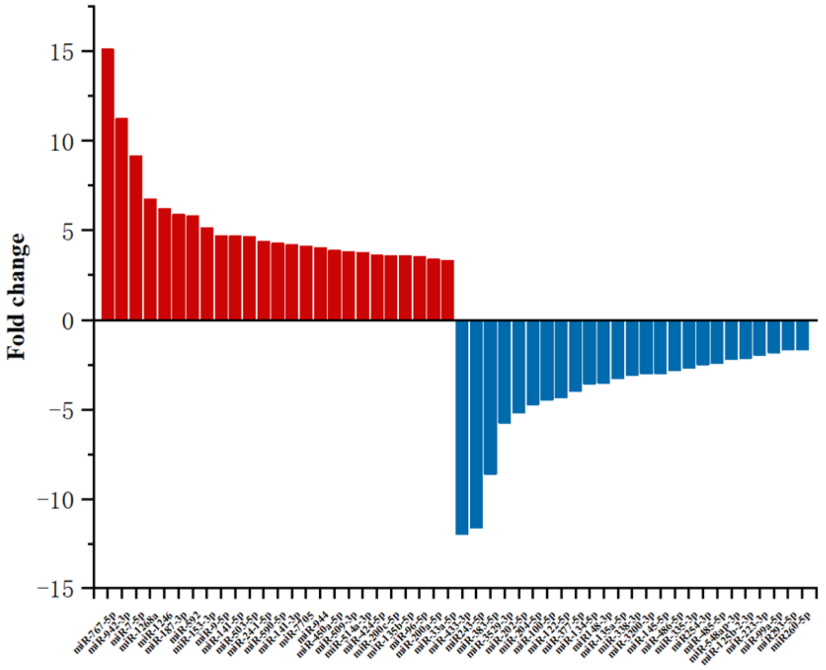

Figure S5. miRNAs expression fold changes in the cSCC samples compared to those of healthy samples. The original data were obtained from RNA sequencing results (data are shown with fold change in top 25 of up-regulated genes and down-regulated genes).

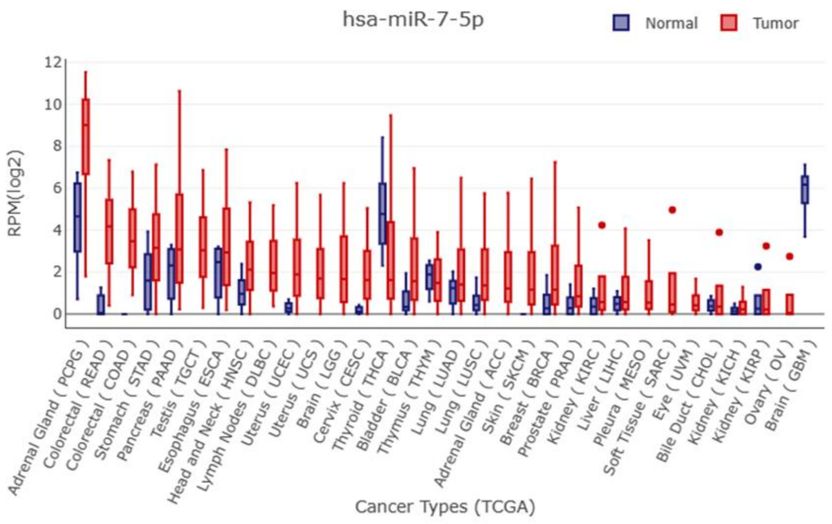

Figure S6. Reads of per million mapped reads (RPM) of miRNA-7 in different organs or tissues of the cSCC samples and healthy samples. The data were obtained from TCGA database.

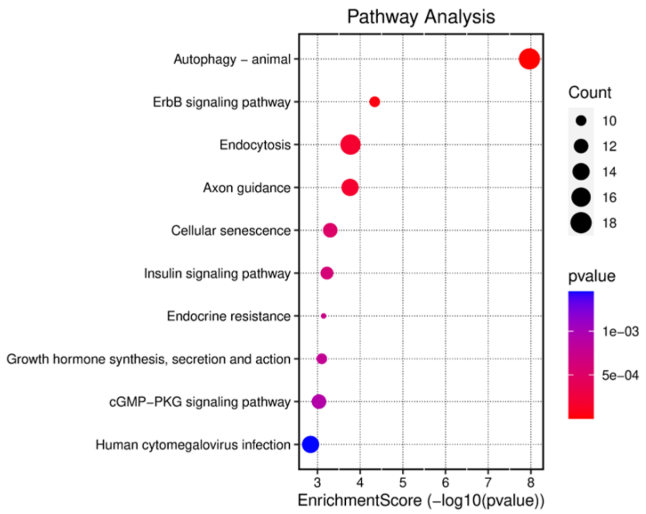

Figure S7. Kyoto Encyclopedia of Genes and Genomes (KEGG) enrichment analysis of miRNA-7 indicated that the signaling pathways of miRNA-7 were mainly associated with autophagy, endocytosis, ErbB signaling pathway, etc.

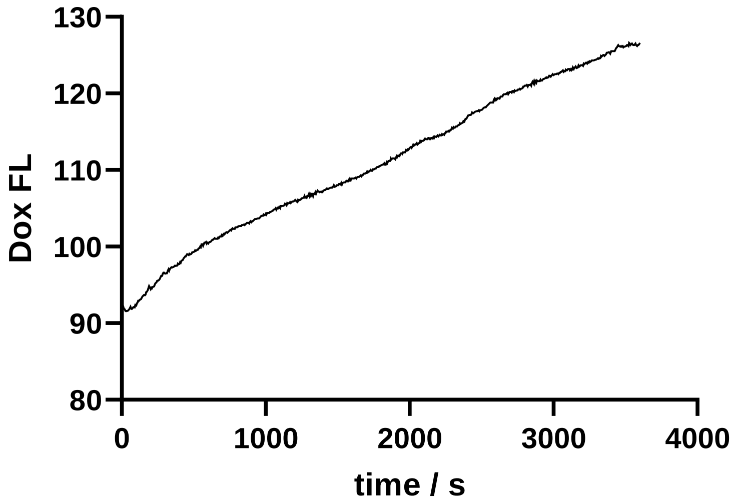

Figure S8. Fluorescence kinetic of free dox showing the controlled release in presence of miRNA-7.

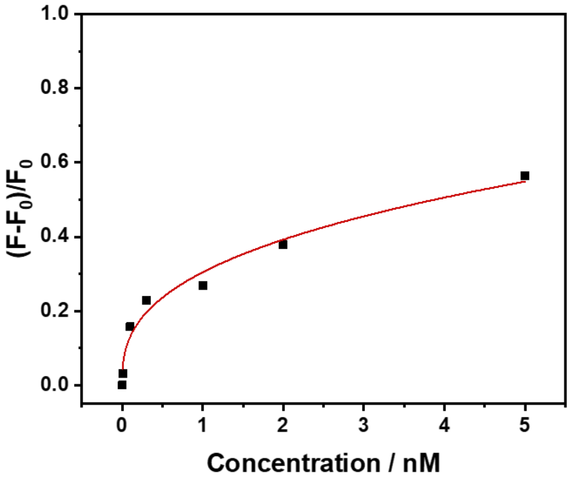

Figure S9. The fitted curve of F-F_0/F_0 ratio was plotted, according to *In vitro* fluorescence profile of TDN responded to different concentrations of complete-matched targets. F_0 was the background signal in the absence of a target, and F was the fluorescence signal in the presence of a target of a certain concentration. The limit of detection was calculated by 3σ/slope calculation.

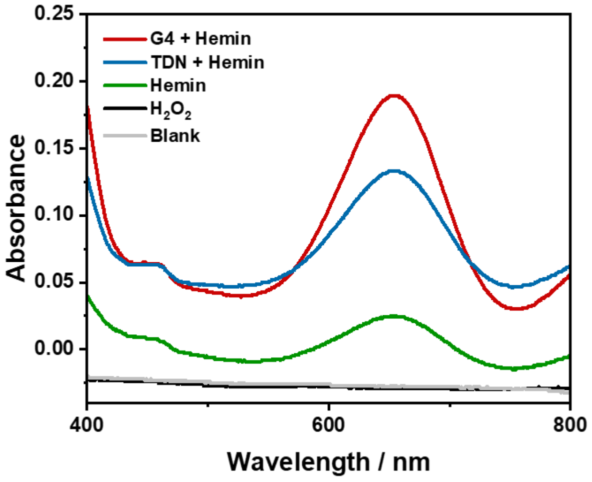

Figure S10. Peroxidase-like catalytic activity of TDN incorporated with Hemin, in the presence of TMB and H_2O_2. The absorbance was recorded by using UV-Vis spectrometer. The G-quadruplex DNA incorporated with Hemin was used as control.

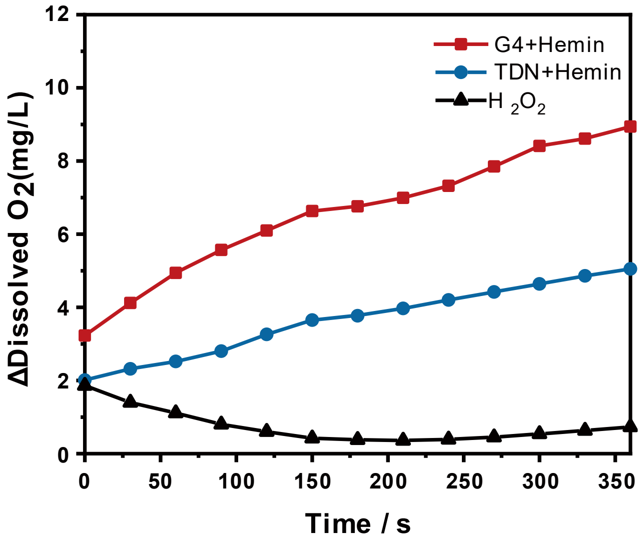

Figure S11. Catalase-like catalytic activity of TDN incorporated with Hemin, in the presence of H_2O_2. Changes in dissolved O2 levels were recorded by a portable dissolved oxygen meter. The G-quadruplex DNA incorporated with Hemin was used as control.

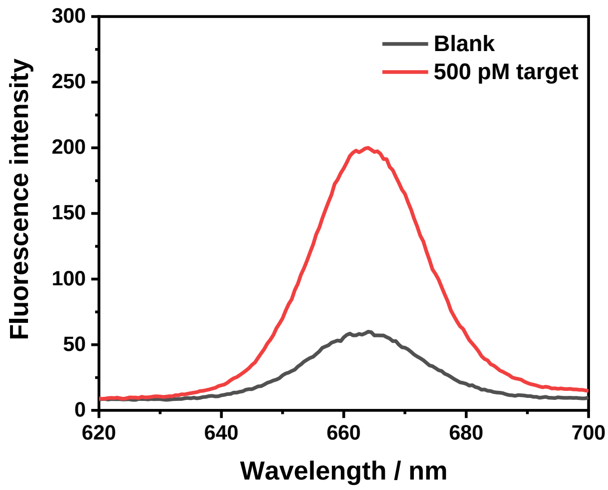

Figure S12. Fluorescence spectrum of ce6-labeled TDN in the absense or presence of 1 nM target.

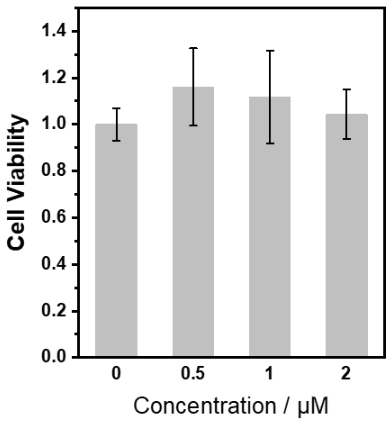

Figure S13. CCK-8 assay to study the biocompatibility of DNA tetrahedral nanostructures. HaCaT cells were incubated with different concentrations (0 μM, 0.5 μM, 1 μM, 2 μM) of DNA tetrahedra for 24 h.

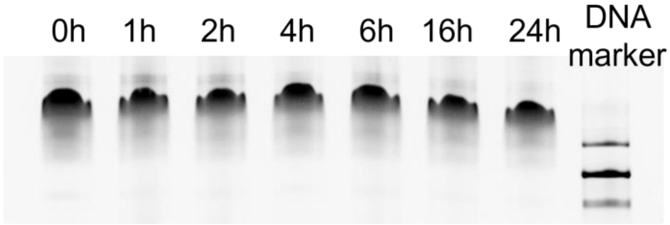

Figure S14. Nuclease resistance analysis of TDN with different incubation time (0 ~ 24 h), by PAGE experiment.

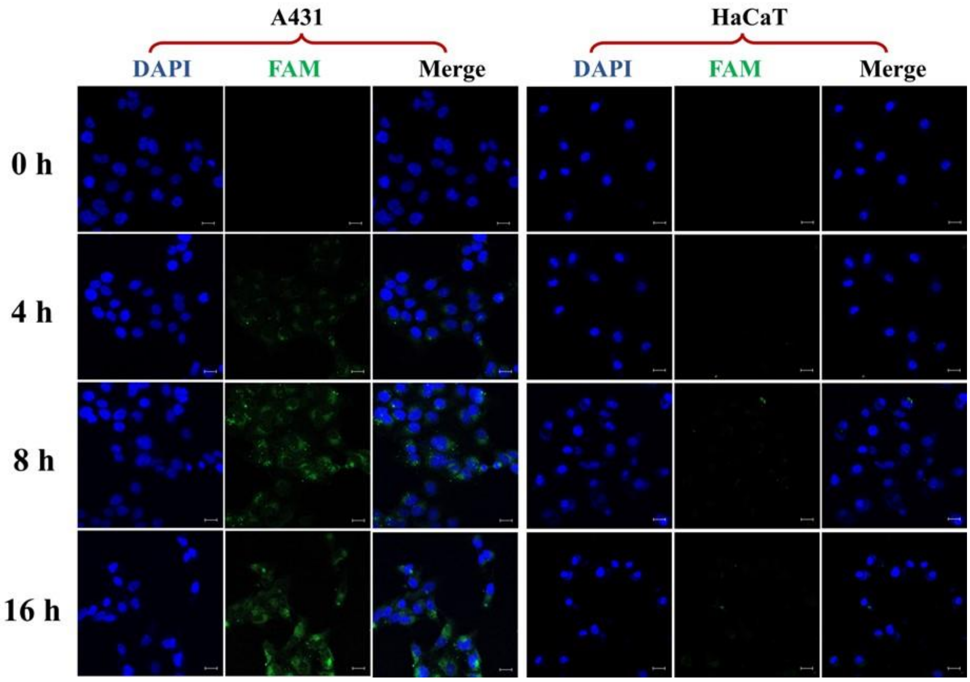

Figure S15. Optimization of cellular uptake and miRNA-responsive fluorescence activation of FAM-labeled TDN in A431 and HaCaT cells. The cells were incubated with 200 nM probes for different time point at 37 °C, followed by confocal microscopy imaging. Scale bars are 20 μm.

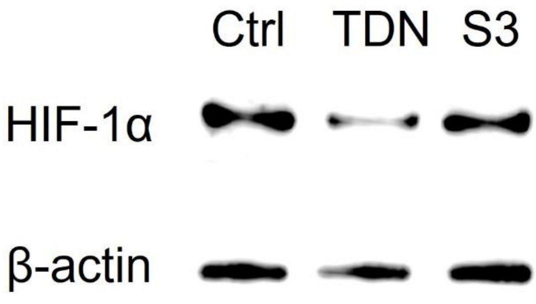

Figure S16. Western blot analysis of HIF-1α expression in cells after different treatments.

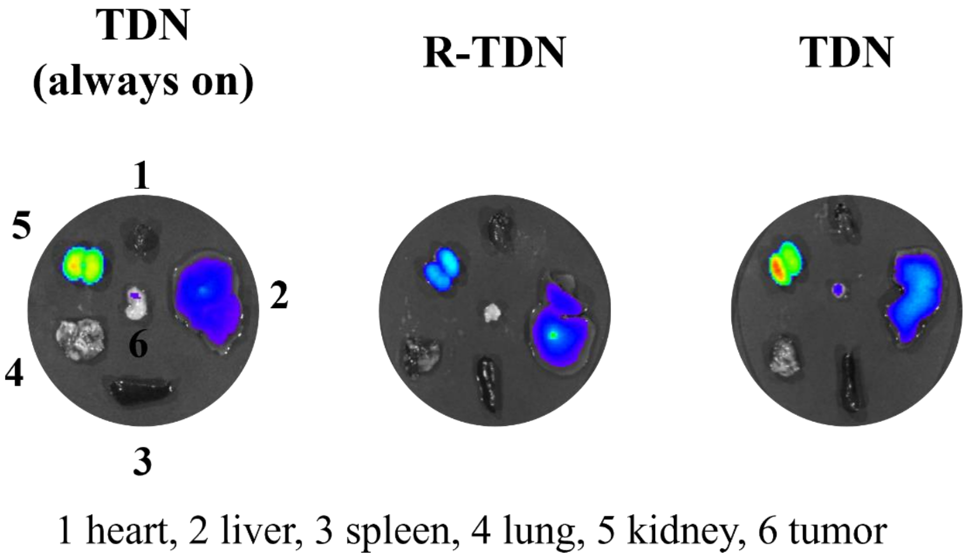

Figure S17. Cy5.5 fluorescence images of the tumor and main organs (including heart, liver, spleen, lung, kidney) harvested from the mice treated with different treatments, after 24 h postinjection.

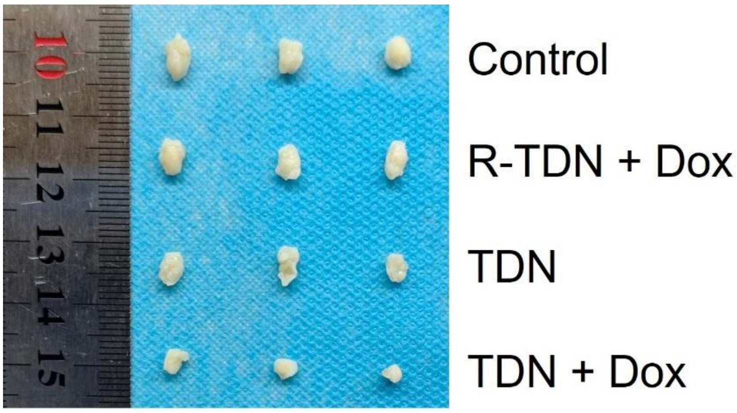

Figure S18. Photos of tumors harvested from mice in each group on day 12. Except for the saline control group, the other groups were irradiated by lasers at the tumor sites with 10 min, after 24 h postinjection.

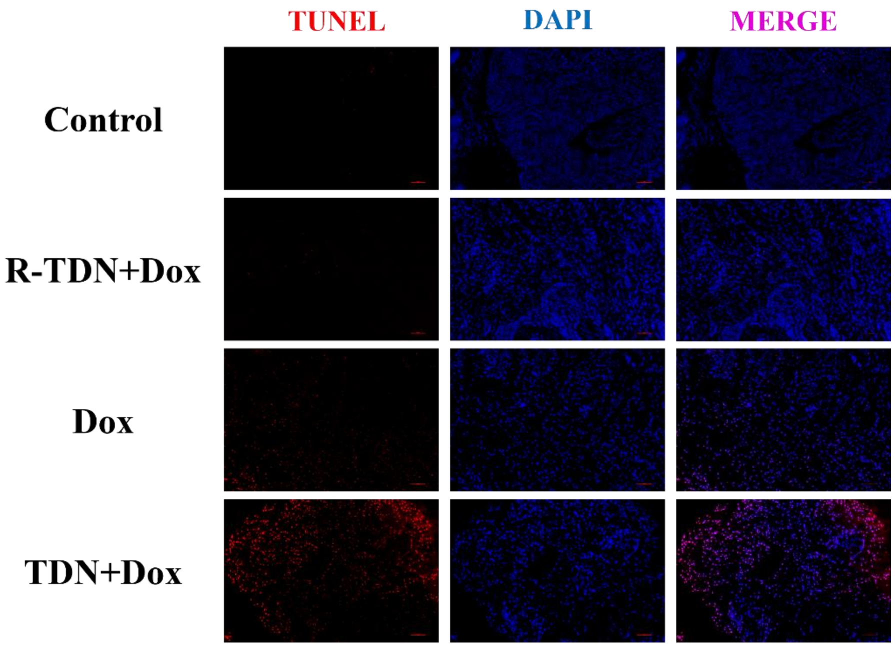

Figure S19. Photos of tumors harvested from mice in each group on day 12. Micrographs of TUNEL stained tumor slices collected from mice 12 days after different treatments. Red and blue signals represent TUNEL-stained apoptotic cells and DAPI-stained cell nuclei, respectively. Scale bars are 100 μm.

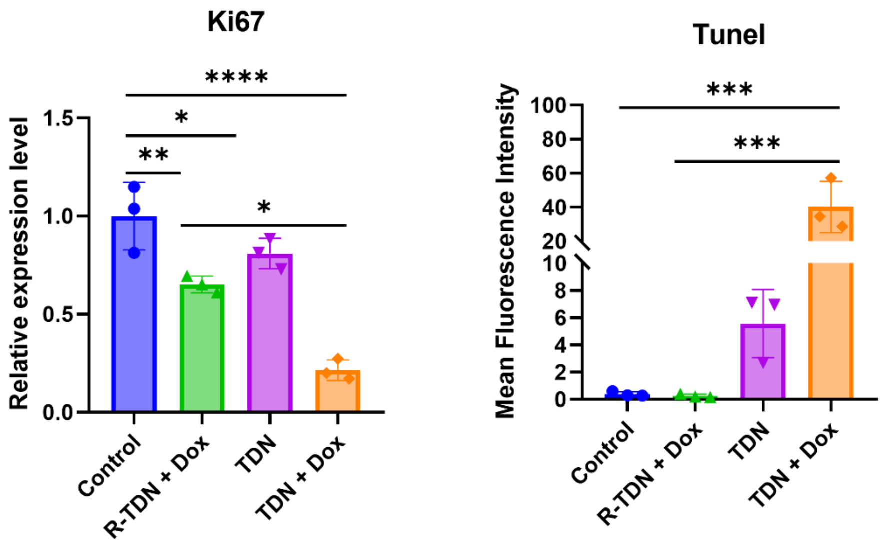

Figure S20. Relative mean fluorescence intensity of Ki-67 (left) and TUNEL (right) immunohistochemical staining results. The quantitative results were normalized with the average fluorescence intensity of the control group.

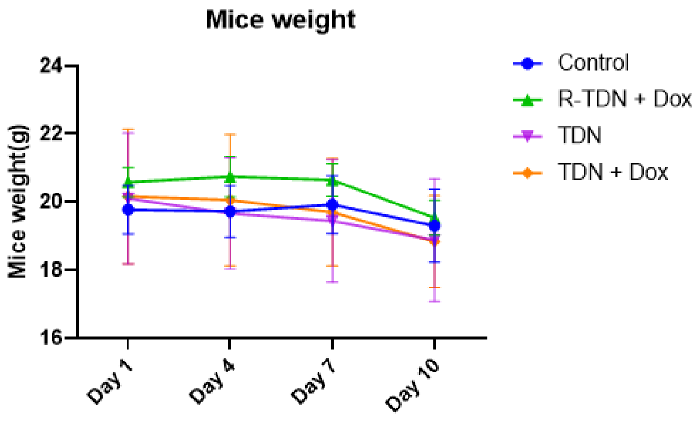

Figure S21. Changes of Mice weight with different treatments. The weight was recorded every three days.

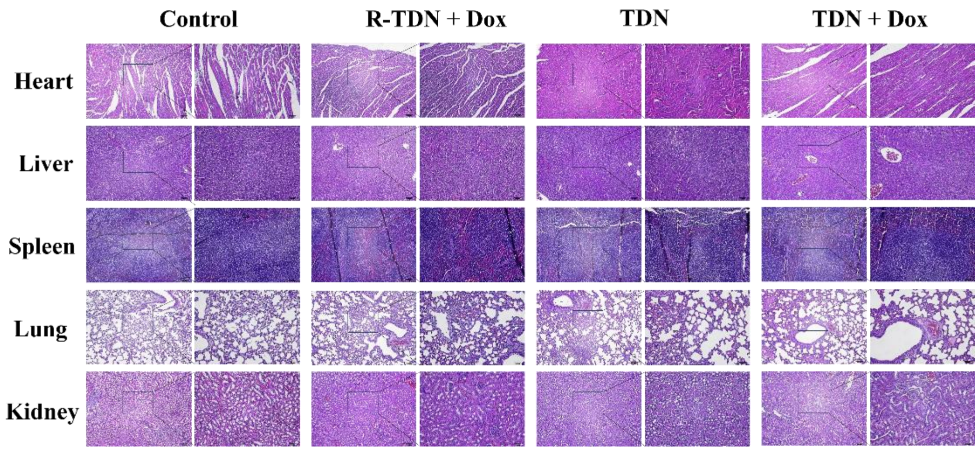

Figure S22. H&E staining observations of the main organs of mice treated with different treatments. There were no significant pathological changes in major organs.

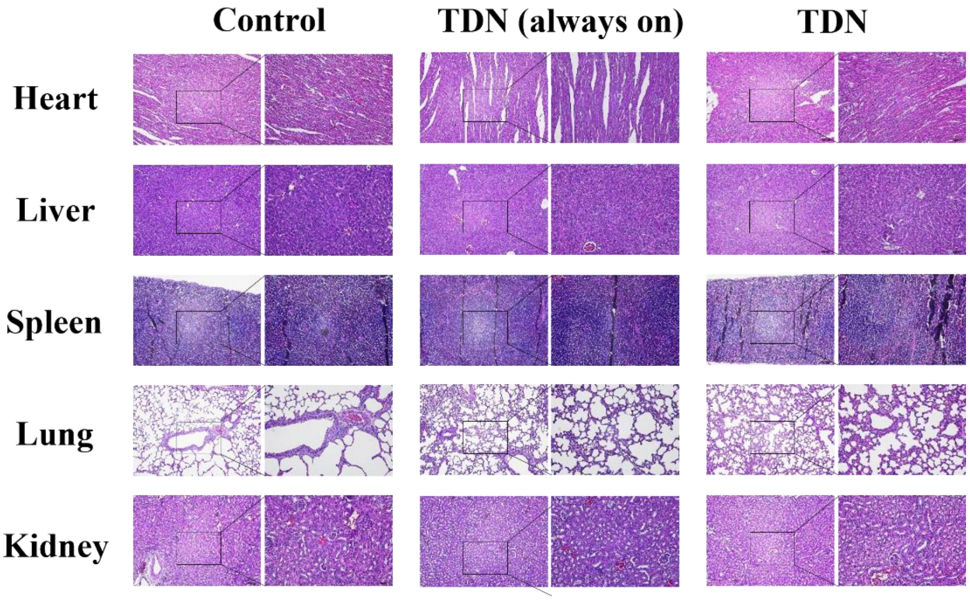

Figure S23. H&E staining observations of the main organs of mice treated with TDN (no BHQ2) and TDN at an equivalent dose of 2.0 nmol of ce6, to study the phototoxicity. There were no significant pathological changes in major organs.

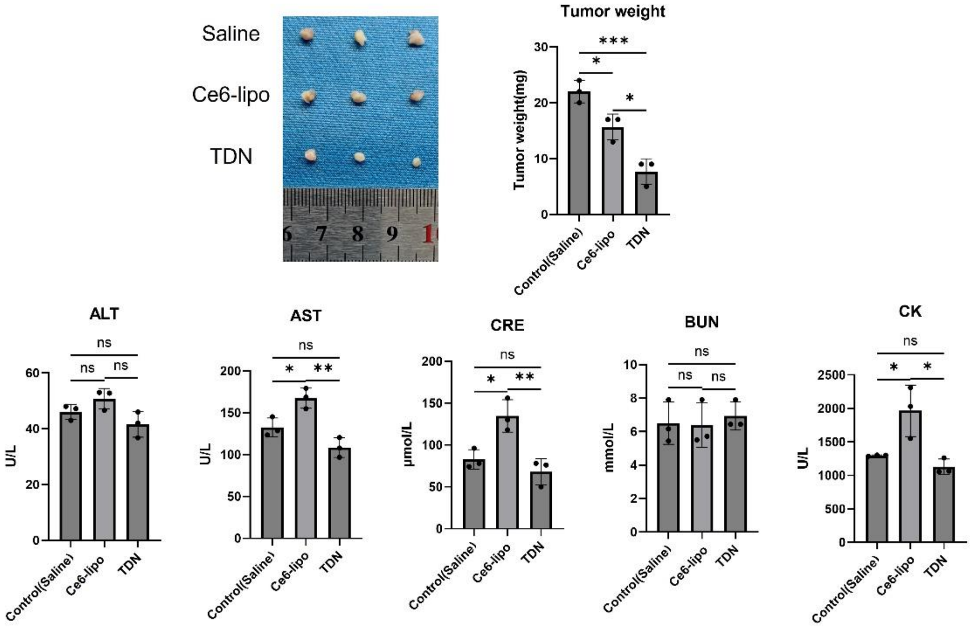

Figure S24. Blood biochemical indexes of ALT, AST, BUN, Cre and CK were detected after different treatments and laser irradiations.

S3 DNA sequence

Table S1. DNA sequences used in this work are listed below.

**Name**	**Sequence (5'- 3')**
S1	TTTATCACCCAAACCCTCAATCTTTTACATTCCTAAGTCTGAA ACATTACAGCTTGCTACACGAGAAGAGCCGCCATAGTA
S1-BHQ2	BHQ2-TTTATCACCCAAACCCTCAATCTTTTACATTCCTAAGTC TGAAACATTACAGCTTGCTACACGAGAAGAGCCGCCATAGTA
S2	TCAGCCAAGCATACTAACTATTTTATCACCAGGCAGTTGACA GTGTAGCAAGCTGTAATAGATGCGAGGGTCCAATAC
S3-AS1411	GGTGGTGGTGGTTGTGGTGGTGGTGGTTTTCAACTGCCTGGT GATAAAACGACACTACGTGGGAATCTACTATGGCGGCTCTTC
S4	TTCAGACTTAGGAATGTGCTTCCCACGTAGTGTCGTTTGTATT GGACCCTCGCAT
H1-FAM	AGATTGAGGGTTTGGGTGATATAACAACAAAATCACTAGTCT TCCACCATGTGTAGATGGAAGACTAGTGAT-FAM
H1-ce6	AGATTGAGGGTTTGGGTGATATAACAACAAAATCACTAGTCT TCCACCATGTGTAGATGGAAGACTAGTGAT-Ce6
H1-Cy5.5	AGATTGAGGGTTTGGGTGATATAACAACAAAATCACTAGTCT TCCACCATGTGTAGATGGAAGACTAGTGAT-Cy5.5
H1	AGATTGAGGGTTTGGGTGATATAACAACAAAATCACTAGTCT TCCACCATGTGTAGATGGAAGACTAGTGAT
R-H1-Cy5.5	AGATTGAGGGTTTGGGTGATTTTTTTTTTTTATCACTAGTCTT CCACCATGTGTAGATGGAAGACTAGTGAT-Cy5.5
H2	TAGTTAGTATGCTTGGCTGATTTGTCTTCCATCTACACATGGT GGAAGACTAGTGATCCATGTGTAGA
miRNA-7	UGGAAGACUAGUGAUUUUGUUGUU
